# Determinants of diet and physical activity (DEDIPAC): a summary of findings

**DOI:** 10.1186/s12966-017-0609-5

**Published:** 2017-11-03

**Authors:** Johannes Brug, Hidde P. van der Ploeg, Anne Loyen, Wolfgang Ahrens, Oliver Allais, Lene F. Andersen, Greet Cardon, Laura Capranica, Sebastien Chastin, Ilse De Bourdeaudhuij, Marieke De Craemer, Alan Donnelly, Ulf Ekelund, Paul Finglas, Marion Flechtner-Mors, Antje Hebestreit, Thomas Kubiak, Massimo Lanza, Nanna Lien, Ciaran MacDonncha, Mario Mazzocchi, Pablo Monsivais, Marie Murphy, Mary Nicolaou, Ute Nöthlings, Donal J. O’Gorman, Britta Renner, Gun Roos, Matthijs van den Berg, Matthias B. Schulze, Jürgen M. Steinacker, Karien Stronks, Dorothee Volkert, Jeroen Lakerveld, on behalf of the DEDIPAC consortium

**Affiliations:** 1 Amsterdam School of Communication Research (ASCoR), University of Amsterdam, Amsterdam, The Netherlands; 2 Department of Public and Occupational Health, Amsterdam Public Health Research Institute, VU University Medical Center, Amsterdam, The Netherlands; 3 Sydney School of Public Health, University of Sydney, Sydney, NSW Australia; 4 Leibniz-Institute for Prevention Research and Epidemiology – BIPS, Bremen, Germany; 5 INRA, UR1303 ALISS, F-94205 Ivry-sur-Seine, France; 6 Department of Nutrition, University of Oslo, Oslo, Norway; 7 Department of Movement and Sports Sciences, Ghent University, Ghent, Belgium; 8 Department of Movement, Human and Health Sciences, University of Rome Foro Italico, Rome, Italy; 9 Institute for Applied Health Research, School of Health and Life Science, Glasgow Caledonian University, Scotland, UK; 10 Centre for Physical Activity and Health Research, University of Limerick, Limerick, Ireland; 11 Norwegian Institute of Public Health, Oslo, Norway; 12 Institute of Food Research, Norwich, UK; 13 Department of Internal Medicine II, Division of Sports and Rehabilitation Medicine, University Ulm, Ulm, Germany; 14 Johannes Gutenberg University, Mainz, Germany; 15 Department of Neurosciences, Biomedicine and Movement Sciences, Verona, Italy; 16 Health Research Institute, University of Limerick, Limerick, Ireland; 17 Department of Statistical Sciences of the University of Bologna, Bologna, Italy; 18 Centre for Diet and Activity Research, MRC-Epidemiology Unit, School of Medicine, University of Cambridge, Cambridge, UK; 19 Sport & Exercise Sciences Research Institute, Ulster University, Northern Ireland, UK; 20 Department of Public Health, Amsterdam Public Health research institute, Academic Medical Centre, University of Amsterdam, Amsterdam, The Netherlands; 21 Department of Nutrition and Food Science, Rheinische Friedrich-Wilhelms-Universität Bonn, Bonn, Germany; 22 3U Diabetes, School of Health and Human Performance, Dublin City University, Dublin, Ireland; 23 Department of Psychology, University of Konstanz, Konstanz, Germany; 24 Consumption Research Norway, Oslo and Akershus University College of Applied Sciences, Oslo, Norway; 25 Department of Prevention & Nutrition, National Institute for Public Health and the Environment, Bilthoven, The Netherlands; 26 Department of Molecular Epidemiology, German Institute of Human Nutrition Potsdam-Rehbruecke, Nuthetal, Germany; 27 Institute for Biomedicine of Aging, Friedrich-Alexander-Universität Erlangen-Nürnberg, Nuremberg, Germany; 28 Department of Epidemiology and Biostatistics, Amsterdam Public Health research institute, VU University Medical Center, De Boelelaan 1089a, 1081 HV Amsterdam, The Netherlands

**Keywords:** Determinants of health behaviours, Dietary behaviour, Europe, Physical activity, Policy evaluation, Sedentary behaviour, Interventions

## Abstract

The establishment of the Determinants of Diet and Physical Activity (DEDIPAC) Knowledge Hub, 2013–2016, was the first action taken by the ‘Healthy Diet for a Healthy Life’ European Joint Programming Initiative. DEDIPAC aimed to provide better insight into the determinants of diet, physical activity and sedentary behaviour across the life course, i.e. insight into the causes of the causes of important, non-communicable diseases across Europe and beyond. DEDIPAC was launched in late 2013, and delivered its final report in late 2016. In this paper we give an overview of what was achieved in terms of furthering measurement and monitoring, providing overviews of the state-of-the-art in the field, and building toolboxes for further research and practice. Additionally, we propose some of the next steps that are now required to move forward in this field, arguing in favour of 1) sustaining the Knowledge Hub and developing it into a European virtual research institute and knowledge centre for determinants of behavioural nutrition and physical activity with close links to other parts of the world; 2) establishing a cohort study of families across all regions of Europe focusing specifically on the individual and contextual determinants of major, non-communicable disease; and 3) furthering DEDIPAC’s work on nutrition, physical activity, and sedentary behaviour policy evaluation and benchmarking across Europe by aligning with other international initiatives and by supporting harmonisation of pan-European surveillance.

## Background

Unhealthy dietary habits, lack of physical activity, and extensive and uninterrupted sitting are known risk factors for major, non-communicable diseases [1]. However, comparatively little is known about the ‘causes of these causes’ of non-communicable disease, i.e. about the most important and modifiable determinants of unhealthy dietary, physical activity, and sedentary behaviour. To further such research, the European Determinants of Diet and Physical Activity (DEDIPAC) Knowledge Hub was established.

In brief, DEDIPAC was the first action taken by the European ‘Joint Programming Initiative (JPI) Healthy Diet for a Healthy Life (HDHL)’ to better align research across Europe in the realm of healthy dietary and physical activity behaviour. DEDIPAC was launched in December 2013 and its mission, purpose, and design were shared with the larger scientific community in a paper published in the *International Journal of Behavioural Nutrition and Physical Activity* in 2014 [2]. DEDIPAC delivered its final report for evaluation and approval on December 1st, 2016.

DEDIPAC’s aim was to understand the determinants, at both the individual and (sub-)population levels, of dietary, physical activity, and sedentary behaviour using a broad, multidisciplinary approach, and to translate this knowledge into more effective promotion of healthy diet and physical activity. To this end, DEDIPAC first aimed to prepare and build the necessary infrastructure for this mission and work towards aligning and coordinating public health research in relation to behaviour in these areas. Today, the DEDIPAC Knowledge Hub is internationally recognised as a network of scientists from various relevant disciplines, and with different levels of seniority, who work together to collect, gain, advance, exchange, and disseminate scientific knowledge and competences in the area of the topic of interest.

The main text of this paper will cover both the methods employed by DEDIPAC, as well as the results of its efforts. We present further detail regarding the infrastructure of the Knowledge Hub and describe how this helped to align research conducted in the context of DEDIPAC, as well as make better use of expertise and available data. Subsequently, we will outline the mix of different methods that were applied to advance the field within the three thematic areas of the Knowledge Hub, and provide an overview of the results of the research conducted in the context of DEDIPAC. In the discussion, we will specifically focus on future steps that may contribute to advancing research on the underlying determinants of major, non-communicable disease within Europe and beyond.

## Methods and results

Joint programming is a process by which European countries define, develop, and implement a common strategic research agenda, based on a shared vision of how to address societal challenges that no country is capable of resolving independently. Such joint programming pools the resources of individual countries such that they can be focused on common goals in an effort to enable more comprehensive, and larger-scale research with more variation in exposure and outcomes. This prevents unnecessary overlap and repetition, thereby enhancing the development and use of standardised research methodology and improving research infrastructure. JPI HDHL’s mission is to enable all Europeans to have the motivation, ability, and opportunity to eat a healthy diet, and undertake sufficient physical activity to contribute to the reduction of the incidence of non-communicable, chronic disease [3].

Twelve countries across Europe (Austria, Belgium, Finland, France, Germany, Italy, Ireland, Norway, Poland, Spain, The Netherlands, and The United Kingdom) agreed to support this first joint action in the context of JPI HDHL, and provided funding via their national governmental funding bodies to support participation and research activities of scientists in their countries. Research groups in the participating countries were then invited to submit expressions of interest in joining an international research consortium dedicated to furthering research on the determinants of diet and physical activity behaviour. Based on these expressions of interest, representatives of the interested research groups were invited to a joint kick-off meeting during which the Knowledge Hub was established, the consortium leadership elected, and a first-draft framework for the proposal of the actual research was outlined. This outline was expanded and developed into a full proposal – entitled, DEDIPAC – over the course of a series of face-to-face and online workshops, meetings, and consultations held over the next three months. This proposal was reviewed and, after reviewers’ comments were taken into account, it was approved by the HDHL management board. DEDIPAC formally started in December, 2013. The final, approved version of the DEDIPAC proposal put forward three key aims:

1. To enable a more standardised and continuous, pan-European ‘needs analysis’, i.e. to monitor dietary, physical activity, and sedentary behaviour, and changes in behaviour in these areas across the life course and across populations, in an effort to identify targets and target populations for (policy) interventions;
2. To explore the main correlates and determinants of these behaviours in and across populations to help to tailor policies and interventions seeking to target these determinants;
3. To learn from the successes and failures of previous and on-going interventions and policies in order to improve evaluation, and increase the effectiveness of future interventions and policies, as well as to identify and benchmark best practices across Europe and compare these internationally.

To work towards realising these aims, DEDIPAC was organised into three Thematic Areas (TAs), and each of these TAs was divided further into work packages (WPs) that, in turn, were broken down into specific tasks:

- TA 1: Assessment and harmonisation of methods for future research, surveillance, monitoring, and evaluation of interventions and policies regarding diet, physical activity, and sedentary behaviour;
- TA 2: Identification of determinants of dietary, physical activity and sedentary behaviour across the life course and in vulnerable groups;
- TA 3: Evaluation and benchmarking of public health interventions and policies aimed at improving dietary, physical activity and sedentary behaviour across the life course.

DEDIPAC was organised as a network, as opposed to having a centrally-led, top-down management structure, and coordinators were elected for both for the overall DEDIPAC organisation, as well as for the individual TAs. The central coordinator and the leaders of the TAs acted as the DEDIPAC management team, mandated to make necessary decisions, subject to the approval of the consortium at large, monitor progress, prepare periodical reports, and report to and align with the JPI HDHL organisation.

**Fig. 1:**
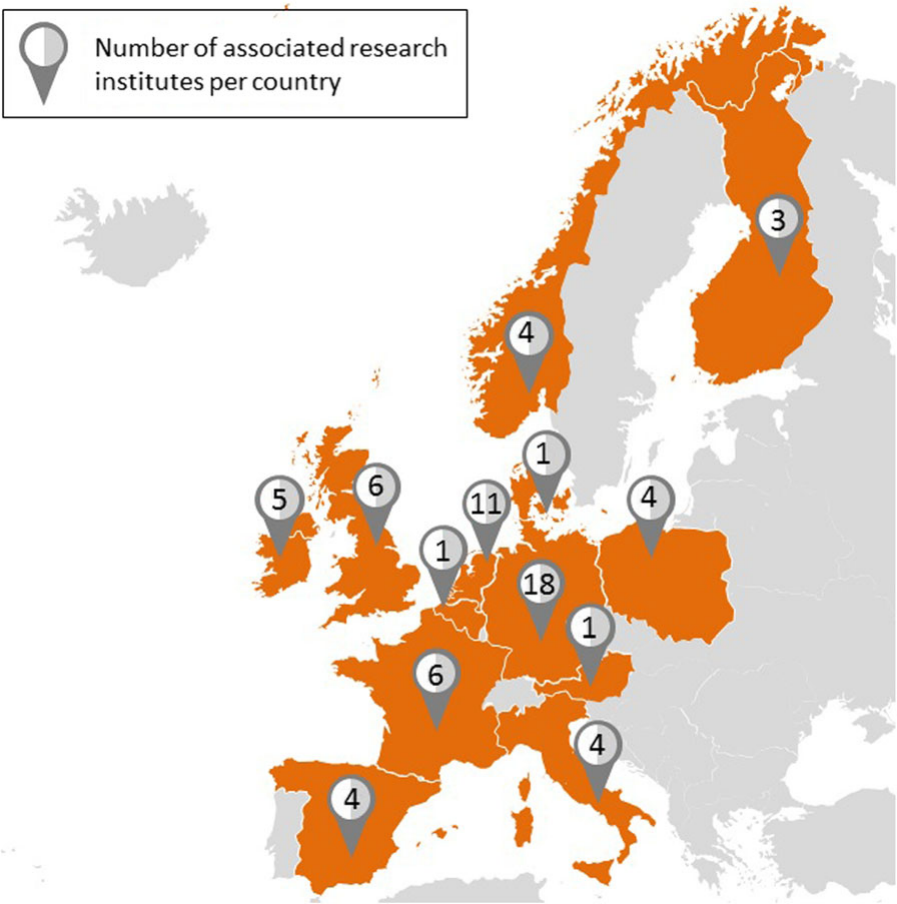
The countries represented in the DEDIPAC Knowledge Hub (in orange) with the number of DEDIPAC institutes per country

In total, almost 300 researchers joined forces as part of the DEDIPAC Knowledge Hub. These individuals hailed from 68 research institutes across all of the participating European countries (see Fig. 1). The Finnish organisations withdrew early in the process, as their national funding body was not able to provide the support necessary to carry out DEDIPAC activities planned by the Finnish partners, and a Danish organisation joined with its own resources *after* the formal establishment of the Knowledge Hub.

DEDIPAC aimed to promote and facilitate knowledge exchange and dissemination and aimed to establish collaborations that would lead to new research and last beyond the initial three-year funding period. A main purpose of DEDIPAC was, thus, to bring together researchers from various relevant disciplines from different countries. This is why it was proposed and presented as a Knowledge Hub, rather than a project or program.

Furthermore, because DEDIPAC was specifically aimed at making the best possible use of available evidence, data, and expertise, the three TAs adopted similar strategies and methodologies that encompassed:

- The provision of methods and a harmonised set of reliable and validated measures to be used for future research, surveillance, and monitoring of the individual, social, and environmental determinants of dietary, physical activity, and sedentary behaviour;
- The provision of state-of-the-art overviews of up-to-date evidence regarding the determinants of these behaviours in different demographic groups by means of series of systematic literature reviews, and mapping/scoping reviews;
- The creation of a level playing field by enabling expert meetings, capacity building, and career development for young researchers, as well as through the creation of integrative frameworks for research;
- Improved use of relevant, existing data through secondary data analyses, including dataset pooling and variable harmonisation;
- Improved dissemination and application of findings and results through the creation of an online platform that provides toolboxes for researchers, practitioners, and policy makers.

Expert consultations with different techniques, such as concept mapping, and some additional, original research complemented these approaches. The literature reviews all complied with the PRISMA guidelines [4], and review protocols were published in PROSPERO [5] where appropriate.

The progress and deliverables have been communicated and disseminated via the DEDIPAC website (https://www.dedipac.eu), scientific publications and non-scientific publications in English and other languages, consortium meetings, as well as in a series of workshops specifically aimed at early-career researchers. DEDIPAC’s output includes 36 papers that have been published or accepted for publication to date, and 34 that are still in progress. These papers include systematic (umbrella) literature reviews, publications on integrative – systems-thinking based – frameworks on determinants of dietary, physical activity, and sedentary behaviour. All currently published DEDIPAC manuscripts are listed in Tables 1, 2, 3 and 4, and DEDIPAC’s open-access output is available via https://www.dedipac.eu. This website also contains the publicly-available and accessible ‘toolboxes’ that are designed to guide and aid researchers, policy makers, and health-promotion professionals in the areas of methodology, identifying determinants, and in exploring intervention and policy best practices. The following section will highlight the TAs’ objectives, methods, and results in more detail.

**Table 1:** Published manuscripts from DEDIPAC Thematic Area 1

Authors	Subject/independent variable	Behaviour/dependent variable	Age group	Study design	Countries	Main conclusions
Surveillance systems						
Bel-Serrat et al. [6]	Surveillance systems	Dietary, physical activity and sedentary behaviour	Across the life course	Inventory	Cross-European	“Many on-going activities were identified at the national level focussing on adults, but fewer surveillance systems involving vulnerable groups such as infants and pre-school children. Assessment of sedentary and dietary behaviours should be more frequently considered. There is a need for harmonisation of surveillance methodologies, indicators and target populations for between-country and over time comparisons. This inventory will serve to feed future discussions within the DEDIPAC-JPI major framework on how to optimize design and identify priorities within surveillance.”
Assessment methods						
Riordan et al. [7]	Assessment methods	Intake of sugar-sweetened beverages	Across the life course	SLR	Cross-European	“The current review highlights the need for instruments to use an agreed definition of sugar-sweetened beverages. Methods that were tested for validity and used in pan-European populations encompassing a range of countries were identified. These methods should be considered for use by future studies focused on evaluating consumption of sugar-sweetened beverages.”
Riordan et al. [8]	Assessment methods	Intake of fruits and vegetables	Across the life course	SLR	Cross-European	“The current review indicates that an agreed classification of fruits and vegetables is needed in order to standardise intake data more effectively between European countries. Validated methods used in pan-European populations encompassing a range of European regions were identified. These methods should be considered for use by future studies focused on evaluating intake of fruits and vegetables.”
Gebremariam et al. [9]	Assessment methods	Availability and accessibility of food	Youth (≤18y)	SLR	International	“The review identified several measures of food availability or accessibility among youth with satisfactory evidence of reliability and/or validity. Findings indicate a need for more studies including measures of accessibility and addressing its conceptualization. More testing of some of the identified measures in different population groups is also warranted, as is the development of more measures of food availability and accessibility in the broader environment such as the neighbourhood food environment.”
Population levels						
Loyen et al. [10]	Variation in population levels	Physical activity	Adults (≥18y)	SLR	Cross-European	“The included studies showed substantial variation in the assessment methods, reported outcome variables and, consequently, the presented physical activity levels. Because of this, absolute population levels of physical activity in European adults are currently unknown. However, when ranking countries, Ireland, Italy, Malta, Portugal and Spain generally appear to be among the less active countries. Objective data of adults across Europe is currently limited. These findings highlight the need for standardisation of the measurement methods, as well as cross-European monitoring of physical activity levels.”
Loyen et al. [11]	Variation in population levels	Sedentary time	Adults (≥18y)	SLR	Cross-European	“One third of European countries were not included in any of the studies. Objective measures of European adults are currently limited, and most studies used single-item self-reported questions without assessing sedentary behaviour types or domains. Findings varied substantially between studies, meaning that population levels of sedentary time in European adults are currently unknown. In general, people living in northern Europe countries appear to report more sedentary time than southern Europeans. The findings of this review highlight the need for standardisation of the measurement methods and the added value of cross-European surveillance of sedentary behaviour.”
Van Hecke et al. [12]	Variation in population levels	Physical activity	Youth (<18y)	SLR	Cross-European	“Reported levels of physical activity and prevalence of compliance to physical activity recommendations in youth showed large variation across European countries. This may reflect true variation in physical activity as well as variation in assessment methods and reported outcome variables. Standardization across Europe, of methods to assess physical activity in youth and reported outcome variables is warranted, preferably moving towards a pan-European surveillance system combining objective and self-report methods.”
Verloigne et al. [13]	Variation in population levels	Sedentary time	Youth (<18y)	SLR	Cross-European	“A substantial number of published studies report on levels of sedentary time in children and adolescents across European countries, but there was a large variation in assessment methods. Questionnaires (child specific) were used most often, but they mostly measured specific screen-based activities and did not assess total sedentary time. There is a need for harmonisation and standardisation of objective and subjective methods to assess sedentary time in children and adolescents to enable comparison across countries.”
Secondary data analysis						
Steene-Johannessen et al. [14]	Agreement between self-report and objective measurements	Meeting the physical activity recommendations	Adults (≥18y)	Secondary, CS	DK, FR, DE, GR, IT, NL, NO, SP, SW, UK	“The modest agreement between self-reported and objectively measured physical activity suggests that population levels of physical activity derived from self-report should be interpreted cautiously. Implementation of objective measures in large-scale cohort studies and surveillance systems is recommended.”
Loyen et al. [20]	Accelerometer pooling	Physical activity and sedentary time	Adults (≥18y)	Secondary, pooled, CS	England, Norway, Portugal, Sweden	“We found high levels of sedentary time and physical inactivity in four European countries. Older people and obese people were most likely to display these behaviours and thus deserve special attention in interventions and policy planning. In order to monitor these behaviours, accelerometer-based cross-European surveillance is recommended.”

**Table 2:** Published manuscripts from DEDIPAC Thematic Area 2

Authors	Subject/independent variable	Behaviour/dependent variable	Age group	Study design	Countries	Main conclusions
Determinant reviews						
Symmank et al. [28]	Determinants	Food decision making	Across the life course	Systematic Inter-disciplinary Mapping (SIM) review	International	“After applying qualitative and quantitative analyses, this study reveals that most of the research [on food decision making] emphasizes biological, psychological, and product related predictors, whereas policy-related influences on food choice are scarcely considered”
Condello et al. [29]	Behavioural determinants	Physical activity	Across the life course	Umbrella SLR	International	“Although the majority of the evidence was limited and most of the determinants were not associated with PA, this umbrella SLR provided a comprehensive overview of the associations between behavioural determinants and PA. Youth should be physically active in the early years and increase active transport to/from school, independent mobility, and ‘free-range activities’ without adult supervision, whilst adult PA behaviours are mostly influenced by the life events. Finally, more research is needed that incorporates prospective study designs, standardized definitions of PA, objective measurement methods of PA assessment, and the use of interactionist and mediational approached for the evaluation of different behavioural determinants influencing PA behaviours.”
Cortis et al. [31]	Psychological determinants	Physical activity	Across the life course	Umbrella SLR	International	“This umbrella SLR provided a comprehensive overview of the associations between psychological determinants and PA. Most of the evidence resulted probable and limited, mainly due to differences in the definition of PA and of psychological determinants across reviews. Convincing evidence was found for a positive association between self-efficacy and PA in children and adolescents, and a negative association between stress and PA regardless of age. At present, there is a need of a consensus on clear definitions of relevant psychological determinants of PA to allow clear interpretations and generalizability of findings. Furthermore, it is envisaged that psychological determinants should be considered within a larger and multi-level framework of determinants to determine possible interactions or mediations of the effects.”
Puggina et al. [32]	Policy determinants	Physical activity	Across the life course	Umbrella SLR	International	“This umbrella systematic literature review summarizes the current evidence on the policy determinants of PA across the life course at individual and population levels. The majority of the reviews resulted of moderate quality. Furthermore, none of the investigated policy determinants had a convincing level of evidence, and very few had a probable level of evidence. At individual level, a clear association between time spent outdoors and PA emerged for children, whereas a limited evidence was found for working hours negatively associated with PA in adults. At the population level, community- and street-scale urban design and land use policies were found to positively support PA levels, although levels of evidence were low. Therefore, further research is needed, preferably by using prospective study designs, standardized definitions of PA and objective measurement of PA.”
Carlin et al. [30]	Physical environment determinants	Physical activity	Across the life course	Umbrella SLR	International	“This umbrella systematic literature review provided a comprehensive overview of the physical determinants of PA across the life course. The limited evidence available from longitudinal studies, coupled with the diverse methodologies and definitions of both PA outcomes and physical determinants/factors employed across studies, makes it difficult to draw firm conclusions. It is vital that researchers make a concerted effort to employ harmonised, objective methodologies in the future measurement of PA and its determinants.”
Stierlin et al. [33]	Determinants	Sedentary behaviour	Youth (<18y)	SLR	International	“Multiple potential determinants were studied in only one or two studies. Determinants were found at the individual, interpersonal, environmental and policy level but few studies examined acomprehensive set of factors at different levels of influences. Evidence was found for age being positively associated with total sedentary behaviour, and weight status and baseline assessment of screen time being positively associated with screen time (at follow-up). A higher playground density and a higher availability of play and sports equipment at school were consistently related to an increased total sedentary behaviour, although these consistent findings come from single studies. Evidence was also reported for the presence of safe places to cross roads and lengthening morning and lunch breaks being associated with less total sedentary behaviour.”
O’Donoghue et al. [34]	Determinants	Sedentary behaviour	Adults (18–65y)	SLR	International	“Results provide further evidence relating to several already recognised individual level factors and preliminary evidence relating to social and environmental factors that should be further investigated. Most studies relied upon cross-sectional design limiting causal inference and the heterogeneity of the sedentary measures prevented direct comparison of findings. Future research necessitates longitudinal study designs, exploration of policy-related factors, further exploration of environmental factors, analysis of inter-relationships between identified factors and better classification of sedentary behaviour domains.”
Chastin et al. [35]	Determinants	Sedentary behaviour	Older adults (>65y)	SLR	International	“Few studies have investigated determinants of sedentary behaviour in older adults and these have to date mostly focused on personal factors, and qualitative studies were mostly lacking. More longitudinal studies are needed as well as inclusion of a broader range of personal and contextual potential determinants towards a systems-based approach, and future studies should be more informed by qualitative work.”
Osei-Kwasi et al. [36]	Determinants	Dietary behaviour	Across the life course; minority groups	SLR	Cross-European	“This review identified a broad range of factors and clusters influencing dietary behaviour among ethnic minority groups. Gaps in the literature identified a need for researcher to explore the underlying mechanisms that shape dietary behaviours, which can be gleaned from more holistic, systems-based studies exploring relationships between factors and clusters. The dominance of studies exploring ‘differences’ between ethnic minority groups and the majority population in terms of the socio-cultural environment and food beliefs suggests a need for research exploring ‘similarities’. The evidence from this review will feed into developing a framework for the study of factors influencing dietary behaviours in ethnic minority groups in Europe.”
Langøien et al. [37]	Determinants	Physical activity and sedentary behaviour	Across the life course; ethnic minority groups	SLR	Cross-European	“Physical activity and sedentary behaviour among ethnic minority groups living in Europe are influenced by a wide variety of factors, especially informed by qualitative studies. More comparative studies are needed as well as inclusion of a larger number of ethnic minority group resettled in different European countries. Few studies have investigated factors influencing sedentary behaviour. It is important in the future to address specific factors influencing physical activity and sedentary behaviour among ethnic minority groups in order to plan and implement effective interventions.”
Determinant frameworks						
Stok et al. [38]	Determinants Of Nutrition and Eating (DONE) framework	Dietary behaviour	Across the life course	Multi-phase, multi-method process	International	“In the creation phase, mind mapping, knowledge mapping, and several discussion and consensus rounds were employed to generate a comprehensive, systematically structured set of determinants of nutrition and eating across the lifespan. In the evaluation phase, priorities for research were determined by rating the determinants on the dimensions of modifiability, relationship strength, and population-level effect. Furthermore, the framework’s quality, usefulness, and comprehensiveness were empirically evaluated by external experts from different disciplines and countries. In the updating phase, a pilot confirmed the feasibility of the continued evolution of the framework by requesting additional input from external experts. Moreover, the framework was dynamically visualized and made freely available on the Internet.”
Condello et al. [39]	European-Physical Activity Determinants (EU-PAD) framework	Physical activity	Across the life course	Concept mapping	Cross-European	“The current framework provides a preliminary overview of factors which may account for physical activity behaviour across the life course and are most relevant to the European community. These insights could potentially be a foundation for future pan-European research on how these factors might interact with each other, and assist policy makers to identify appropriate interventions to maximise physical activity behaviours and thus the health of European citizens.”
Chastin et al. [40]	Systems Of Sedentary behaviour (SOS) framework	Sedentary behaviour	Across the life course	Concept mapping	International	“Through an international transdisciplinary consensus process, the SOS framework was developed for the determinants of sedentary behaviour across the life course. Investigating the influence of Institutional and Home Settings was deemed to be the most important area of research to focus on at present and potentially the most modifiable. The SOS framework can be used as an important tool to prioritise future research and to develop policies to reduce sedentary time.”
Secondary data analysis						
Stelmach-Mardas et al. [45]	Seasonality	Food and energy intake	Adults (≥18y)	SLR	International	“The winter or the post-harvest season is associated with increased energy intake. The intake of fruits, vegetables, eggs, meat, cereals and alcoholic beverages is following a seasonal consumption pattern and at least for these foods season is a determinant of intake.”
Schoen et al. [46]	Notified risk of type 1 diabetes	Dietary quality	Youth (<18y)	Secondary, pooled, CS	Germany	“Nutrient and food intake quality were lower at nine months of age and food intake quality was lower at 24 months of age in at-risk [for type 1 diabetes] than in not-at-risk children (*p* = 0.01 and *p* < 0.0001, respectively). The amount of added sugar was higher in at-risk children at both ages (*p* < 0.0001). In at-risk children, dietary quality was similar between children who were first exposed to gluten at six or 12 months of age. Despite being notified about their child’s risk of T1D, the child’s mother did not switch to healthier diets compared with not-at-risk mothers.”
Wittig et al. [47]	Sex, age, BMI, SES and diet quality	Energy and macronutrient intake	Adults (≥18y)	Secondary, 7CS	Germany	“The presented analyses provide comprehensive descriptions of meal patterns in regard to the distribution of energy intake over the course of the day of selected population groups in Germany. With few differences within the population groups defined by sex, age, BMI, SES, and HEI-NVS-II, the traditional three-main-meal pattern was observed, a result which is also found in other studies. For old adults, meals have an important role for structuring the day as seen in distinct peaks at the three-main-meal periods. In contrast, young adults seem to have a higher variability in energy intake and a less distinct meal pattern. Further, the results show that the highest energy intake was observed in the ‘evening’ period, especially in young adults, overweight persons, and persons with a high SES, as well as men with a low dietary quality (expressed by HEI-NVS-II). Because a high energy intake in the ‘evening’ period is associated with health-related factors, such as obesity, higher hypertension prevalence, and a higher blood pressure, in the literature, the distribution of energy intake over the course of the day should be considered by recommendations for the promotion of a healthy nutritional behaviour.”
Si Hassen et al. [48]	Socioeconomic indicators	Nutrient intake	Adults (≥18y)	Secondary, CS	France	“Low educated participants had higher protein and cholesterol intakes and lower fibre, vitamin C and beta-carotene intakes. Low income individuals had higher complex carbohydrate intakes, and lower magnesium, potassium, folate and vitamin C intakes. Intakes of vitamin D and alcohol were lower in low occupation individuals. Higher income was associated with higher intakes of fibre, protein, magnesium, potassium, beta-carotene, and folate among low educated persons only, highlighting effect modification. Lower SEP, particularly low education, was associated with lower intakes of nutrients required for a healthy diet. Each socio economic position indicator was associated with specific differences in nutrient intake suggesting that they underpin different social processes.”
Gebremariam et al. [49]	Screen-based sedentary time	Soft drink consumption	Youth (<18y)	Secondary, CS	International	“TV viewing appears to be independently associated with soft drink consumption and this association was moderated by parental education in two countries only. Reducing TV time might therefore favorably impact soft drink consumption.”
Totland et al. [50]	Correlates	Irregular family meal patterns	Youth (<18y)	Secondary, CS	Cross-European	“The majority of 11-year-old children regularly ate breakfast and dinner with their families. More television viewing and less vegetable consumption were associated with irregular family breakfasts and dinners, respectively. Social differences were observed in the regularity of family breakfasts. Promoting family meals across social class may lead to healthier eating and activity habits, sustainable at the population level.”
Lakerveld et al. [51]	Correlates	Sedentary behaviour	Adults (≥18y)	Secondary, CS	Cross-European	“Higher socio-economic status subgroups were generally more likely to sit for extended time as compared to people with a lower socio-economic status. Type of occupation was the primary discriminator. In addition, gender, level or urbanization and internet use were important predictors of sitting >7.5 h/day. Gender differences depended on the specific context.”
Loyen et al. [52]	Correlates	Sedentary behaviour	Adults (≥18y), ethnic minority groups	Secondary, CS	Netherlands	“No statistically significant differences in the levels of objectively measured sedentary time or its socio-demographic and lifestyle-related correlates were observed among five ethnic groups in Amsterdam, the Netherlands.”

**Table 3:** Published manuscripts from DEDIPAC Thematic Area 3

Authors	Subject/independent variable	Behaviour/dependent variable	Age group	Study design	Countries	Main conclusions
Quality of policies and interventions						
Horodyska et al. [56]	Good practice characteristics of interventions and policies	Dietary, physical activity and sedentary behaviours	N/A	Umbrella review	International	“The use of the proposed list of 53 good practice characteristics may foster further development of health promotion sciences, as it would allow for identification of success vectors in the domains of main characteristics of interventions/policies, their implementation, evaluation and monitoring processes.”
Implementation and transferability						
Horodyska et al. [57]	Evidence-based conditions important for successful implementation of interventions and policies	Dietary, physical activity and sedentary behaviours	N/A	Umbrella review	International	“The use of the proposed list of 83 conditions for successful implementation may enhance the implementation of interventions and policies which pursue identification of the most successful actions aimed at improving diet, physical activity and reducing sedentary behaviours.”

**Table 4:** Other published manuscripts from DEDIPAC

Authors	Title
Brug and Chinapaw [63]	Determinants of engaging in sedentary behavior across the lifespan; lessons learned from two systematic reviews conducted within DEDIPAC
Chastin et al. [64]	Development of a Consensus Taxonomy of Sedentary Behaviors (SIT): Report of Delphi Round 1
Lakerveld et al. [43]	Identifying and sharing data for secondary data analysis of physical activity, sedentary behaviour and their determinants across the life course in Europe: general principles and an example from DEDIPAC

### Objectives, methods and results of TA1

The overall objective of TA1 was to provide the pan-European research community with a harmonised set of reliable and valid measurement methods to be used for future research on dietary, physical activity, and sedentary behaviour and their individual, socio-cultural, and environmental determinants. This objective was translated in four specific goals:

1. To provide an overview of the state-of-the-art in assessment methods and tools in the areas of dietary, physical activity, and sedentary behaviour and make these available in an online toolbox;
2. To provide an overview of the currently available data on dietary, physical activity, and sedentary behaviour and their determinants across Europe;
3. To provide an inventory of state-of-the-art surveillance systems (national, regional, and international) in Europe that assess dietary, physical activity and sedentary behaviours, and to identify gaps in current systems, methods, and tools and formulate recommendations on how to fill these gaps;
4. To develop a roadmap for a state-of-the-art, harmonised, pan-European surveillance system of dietary intake, dietary behaviour, physical activity, and sedentary behaviour and their key determinants, with a focus on children and adolescents. This subgroup was chosen as current surveillance systems do not allow for longitudinal and regional comparisons of the prevalence of overweight/obesity in children and adolescents, the related lifestyle behaviour, and the key determinants of this behaviour. This prioritisation was based on a needs and gaps analysis of the DEDIPAC inventory on existing surveillance systems in Europe [6].

TA1 was further broken down into two WPs that addressed the assessment and harmonisation in the areas of dietary intake and dietary behaviour (WP1.1), and physical activity and sedentary behaviour (WP1.2). A third WP focused on pan-European harmonisation of research and surveillance regarding dietary and physical activity behaviour and their determinants (WP1.3). Harmonisation, in this respect, refers to the process of minimizing differences in measures, variables, and methods, so that outcomes are comparable.

#### Overview of the state-of-the-art and identification of gaps

For goals 1–3, 19 systematic literature reviews were performed to provide an overview of the state-of-the-art in terms of assessment methods and tools. It was shown that dietary behaviour is usually assessed using food frequency questionnaires (FFQs) in etiologic research and 24-h dietary recalls or food record methods in the context of surveillance. These are self-reported, but standardised methods for which validity has been evaluated at the level of selected nutrients using advanced measurement-error models. Over the course of the project, several systematic reviews identified specific dietary assessment tools and methods used in existing pan-European studies to assess dietary intake and behaviour in terms of food (e.g. consumption of sugar-sweetened beverages [7] and fruits and vegetables [8]) and availability and accessibility of food [9], nutrient intake, dietary patterns, and meal patterns.

The systematic assessment of currently available data on the prevalence of physical activity and sedentary behaviour and its determinants across Europe was described in a set of four reviews [10–13] that revealed that physical activity and sedentary behaviour are assessed using a range of methods and measures, most commonly including self-report questionnaires, and less often, the monitoring of wearable technology, including pedometers, heart-rate monitors, accelerometers, inclinometers, or combined sensors. The majority of methodological studies have found that these worn devices are increasingly available, provide increased validity, reliability, and sensitivity, and are therefore potentially useful in terms of improving comparability of surveillance systems across Europe. Conversely, self-reporting tends to overestimate physical activity [14]. Nevertheless, self-reported data still adds essential contextual information, and thus may be used to supplement data extracted from worn devices. In addition, although accelerometers, or other such devices, provide more objective assessment of physical activity and sedentary behaviour, decisions related to data management and analysis still remain somewhat subjective. As such, we do not claim that the use of such devices eliminates subjectivity.

In terms of the upstream determinants of this behaviour, the availability of standardised assessment methods lags behind, and knowledge of individual-level psychosocial determinants is largely based on self-reporting, thus limiting international comparability. Pan-European standardisation of methods and instruments is lacking, which also results in data being largely incomparable.

The state-of-the-art assessment methods for dietary, physical activity, and sedentary behaviour and determinants that were identified in the systematic literature reviews were summarised in a publicly available, online toolbox that includes information on the validity, reliability, and acceptability (perceived by participants) of the assessment methods. The toolbox can be found here: https://www.dedipac.eu/toolbox/. Additionally, analyses of secondary data were conducted using existing datasets (e.g. the Attitude Behaviour Change study [15]; the Swedish Neighborhood and Physical Activity study [16]; the Health Survey for England [17]; a Norwegian physical activity prevalence study [18]; and a Portuguese physical activity prevalence study) [19]. The systematic literature reviews and secondary data analyses provided an overview of the state-of-art, and helped identify major gaps with regard to methodology and availability of data on prevalence and determinants, as well as geographical blind spots.

For physical activity and sedentary behaviour, the studies included in the systematic literature reviews of population levels showed substantial variation in the assessment methods, reported outcome variables, and, consequently, the reported physical activity levels and time spent sedentary [10–13]. Because of this, absolute population levels of physical activity and time spent sedentary in European youth and adults are currently largely unknown. Hence, there is a need for harmonisation and standardisation of methods to assess these behaviours and to enable better comparison across European countries. The pooling of accelerometer data in population-based studies provided some data allowing comparisons between countries that revealed, for example, that the most active countries can also be the most sedentary countries [20]. This latter combination is possible within a population, but also for an individual (as one may meet the physical activity guidelines on a particular day, but may also remain sedentary for a great deal of time on the same day). Nevertheless, pooling of accelerometer data does have its own challenges, and the availability of accelerometer data in population-based samples is still very limited (only four European countries have such data for population-based, adult samples).

Finally, an initial step toward bridging the gaps that were identified in assessment methodologies was made by developing and evaluating a new research instrument that assesses sugar-sweetened beverage consumption, as well as a novel instrument that can be used to assess sedentary behaviour and media use for surveillance purposes – both relevant, important, and under-researched topics regarding health behaviour, particularly in young populations. Appropriate existing questionnaires were screened and, where suitable, some of their components were adapted and integrated into the new instrument. This was complemented by a manual containing a Standard Operating Procedure. Another selected and adapted instrument was a smartphone-based method of assessing sugar-sweetened beverage consumption, and an inclinometer to assess physical activity and sedentary behaviour in young adults. The novel instruments were pilot tested in multiple countries.

#### Development of the roadmap for a harmonised pan-European monitoring system

An inventory was made of national and international European surveillance systems covering diet, physical activity, and sedentary behaviour, and determinants of health behaviour [6]. Based on this inventory, we observed that children and adolescents followed by elderly people were the age groups that were least well-covered by current surveillance systems. We, therefore, prioritised a roadmap toward a harmonised pan-European surveillance system targeting youth. In particular, sedentary behaviour emerged as the domain that was least assessed in children and adolescents, followed by physical activity. In addition, the European Strategy Forum on Research Infrastructures-Biological and Medical Science Research Infrastructures (ESFRI-BMS RIs) was approached to explore potential synergies between the European RI landscape and DEDIPAC. Working toward a stepwise implementation, we identified currently existing surveillance systems that provide state-of-the-art instruments, or that provide a pan-European infrastructure that could potentially serve as a harmonised surveillance system. Six international surveillance systems were selected as key initiatives: the WHO European Childhood Obesity Surveillance Initiative (COSI) [21]; the Health Behaviour in School-aged Children (HBSC) study [22]; the EU Menu project (aiming to provide standardised information on what people eat in all countries and regions across the EU) [23]; GloboDiet (working towards adapting a standardised international 24-h dietary recall methodology) [24]; European Health Interview Survey [25]; and the Nordic Monitoring of Diet, Physical Activity and Overweight [26]. The German Health Interview and Examination Survey for Children and Adolescents (KiGGS) initiative was selected to serve as a model for the implementation of objective measurement methods [27]. These initiatives contributed to the roadmap.

During an expert meeting, concrete action steps were formulated for the roadmap that were laid down in a conceptual framework. Six action steps with regard to the conceptual framework for implementing the roadmap were proposed: 1) key indicators for dietary intake, dietary behaviour, physical activity, sedentary behaviour, and their determinants should be identified; 2) suitable instruments to assess key indicators across existing surveillance systems should be selected and should a) be valid with the greatest overlap across existing systems; b) prioritise objective measurements over self-reports where feasible; c) use instruments that are robust and easy to apply at a reasonable cost; 3) additional in-depth measurements may be identified and added as optional supplementary modules, e.g. using objective methods in subsamples; 4) a first set of key indicators may be measured using short screening instruments (screeners); 5) the latter could be developed and then implemented by a few select surveillance systems that will superimpose them onto their established instruments as a first step; 6) the stepwise implementation of further screeners may then lead to a gradual replacement of the original, non-harmonised measures, and the successive introduction of newer, and more valid measurements. Methodological studies need to accompany the development, pilot-testing, and implementation of each new module, as well as the calibration of existing instruments.

### Objectives, methods and results of TA2

The overall objective of TA2 was to explore the main correlates and determinants of dietary, physical activity, and sedentary behaviour across the life course, and to help to tailor policies and interventions to target these determinants. This objective was specified in two main goals:

1. To review the current state-of-the art, and develop dynamic and evolving frameworks to guide research on the determinants of dietary, physical activity, and sedentary behaviour;
2. To conduct secondary data analyses that contribute to new knowledge to further develop the frameworks on determinants of dietary, physical activity, sedentary behaviour and social inequality.

To realise these goals, TA2 formed four WPs addressing dietary behaviour (WP2.1), physical activity (WP2.2), sedentary behaviour (WP2.3), and social inequality and ethnic minorities (WP2.4). The first three WPs covered all age groups across the life course, with a primary focus on the general population, while WP2.4 had a specific focus on high-risk populations: groups with a lower socioeconomic position, and ethnic minority and immigrant groups, in particular.

#### Reviewing determinants and developing determinant frameworks

Overall, in the context of TA2, 21 systemic reviews were conducted or are in progress, of which ten have been published at this time: 1 on determinants of diet [28]; 4 on determinants of physical activity [29–32]; 3 on determinants of sedentary behaviour [33–35]; and 2 on ethnic minorities [36, 37] (see Table 2 for the published papers).

For diet, a systematic interdisciplinary mapping (SIM) review of consumer food-decision making and its determinants was conducted using rapid review techniques and the Determinants Of Nutrition and Eating (DONE) framework to explore the state-of-the-art and to identify hot topics and research gaps in this field [28]. This SIM review included 1820 publications from more than ten disciplines (including nutritional science, medicine/health science, psychology, food science and technology, business research, etc.) across a period of 60 years. After applying qualitative and quantitative analyses, this study revealed that most of the research conducted and published to date focused on biological, psychological, and/or product-related predictors, whereas ‘upstream’ influences (e.g. related to policy or built and social environments) on food choice are scarcely considered. In this way, the SIM study highlighted newly identified determinants for future empirical research and showed how measurement of known determinants should be embedded in new or different contexts in future studies (e.g., to embed policy determinants in studies with a focus on individual decision making). A further 8 systematic literature reviews were performed that dealt with determinants of dietary behaviour in different age groups (for the published papers see Table 2).

The results of four of the seven systematic umbrella reviews (‘reviews of reviews’) focusing on the biological, behavioural [29], psychological [31], built environmental [30], socio-cultural, economic, and policy determinants [32] have been published and are summarised in Table 2. In general, the current evidence showed to be of moderate quality. A need for consensus on clear definitions of physical activity and its possible relevant determinants emerged. Furthermore, to allow for clear interpretation and generalisability of findings, determinants of physical activity should be studied within a large and multi-level framework to account for interacting and mediating factors. Finally, further prospective study designs, objective measurement of physical activity, and cohort studies are strongly recommended.

WP2.3 conducted three systematic reviews on determinants of sedentary behaviour across three age groups: youth (<18 years) [33]; adults (18–65 years) [34]; and older adults (>65 years) [35]). The studies included were predominantly conducted in and covered populations from Europe, the US, and Australia. The operationalisation of sedentary behaviour in most studies was limited to TV or ‘screen’ time, rather than overall sedentary behaviour, and often relied on self-reporting. Furthermore, the systematic literature reviews revealed a lack of studies using qualitative research methodologies, as well as a lack of studies that looked into the more motivational and, as was the case in the reviews dealing with determinants of dietary behaviours, the contextual or ‘upstream’ potential determinants of sedentary behaviour.

WP2.4 conducted two systematic mapping reviews of the determinants of diet [36], and physical activity/sedentary behaviour [37] in ethnic minority and migrant origin populations in Europe. There were few large-scale, epidemiological studies, and much of the evidence presented was obtained from qualitative studies. Both reviews identified a broad range of factors and clusters influencing diet and physical activity behaviour among minority ethnic groups, but overall there was a predominance of studies exploring ‘differences’ between minority ethnic groups and the majority population. The studies were mainly conducted in Northern European countries with populations of South Asian origin most often being the object of study. The reviews indicated that there are several gaps in the literature related to the minority populations studied, the countries in which the studies were conducted, the paucity of comparative studies, and lack of attention to sedentary behaviour. In addition, there is a need for interdisciplinary studies to map the interrelationships between different types of determinants (e.g. physical and political environment). Given the diversity of ethnic minorities in many European countries, there is a need for research exploring ‘similarities’, i.e. the relative importance of factors influencing behaviour in the general population, *as well as* in ethnic minority populations.

The results of these reviews informed the first steps toward the development of three behaviour-specific frameworks for determinants of dietary behaviour [38], physical activity [39], and sedentary behaviour [40]. In addition, WP2.4 developed its own frameworks for determinants of dietary behaviour, and physical activity/sedentary behaviour of ethnic minorities, while also further contributing to the three behaviour-specific frameworks. The other high-risk group in WP 2.4, namely lower socio-economic groups, were covered in the general frameworks. The frameworks were all developed over multiple iterations and fostered multidisciplinary systems thinking in an effort to extend the contents of the framework beyond silos of disciplines and existing ecological models. Structured consensus protocols based on concept mapping were used in those dealing with physical activity and sedentary behaviour, and these behaviours among ethnic minorities [41]. Concept mapping is a standardised mixed method that combines qualitative points of view with multivariate statistical analysis to enable a group to gather and organise ideas into a conceptual framework. This involved five main phases: 1) Preparation included the definition of behavioural outcomes, and the creation of a protocol for structuring and standardising the whole process, which detailed the ways in which participants could contribute; 2) a Delphi-like process followed with the objective of compiling an exhaustive list of all potential determinants based on evidence provided by literature reviews and expert judgements; 3) the potential determinants were then structured into groups/systems; 4) next, the potential determinants were ranked and sorted according to research priority, modifiability, and potential population impact; and 5) determinants were visualised as clusters or systems. The ‘data’ generated over the course of these five phases - aggregated from individual expert contributions and multiple consensus events - were processed and visualised. This was done using *Tableau* in WP2.1 (www.tableau.com; Web link) and *Ariadne* in WP2.2 and WP2.3 (www.minds21.org Web link for WP2.2, Web link for WP2.3) software.

For potential determinants of dietary behaviour, the DONE framework was developed [38]. This framework is organised according to three main outcome categories: food choice, eating behaviour, and dietary intake/nutrition. The framework includes 441 determinants and is visualised on an interactive website: https://www.uni-konstanz.de/DONE/. The website allows the user to select determinants by level, including 4 main levels (individual, interpersonal, environment, policy), 11 stem categories (e.g., biology, psychological), and 51 leaf-categories, as well as by different age groups and relevance for ethnic minorities. For all determinants, the degree of modifiability, relationship strength with the respective outcome category, and population-level effect according to expert ratings is visualised. A first systematic interdisciplinary mapping (SIM) review on consumer food decision making using the DONE framework for categorising the identified predictors of food decision making revealed that most of the research emphasises biological, psychological, and product-related predictors, whereas policy-related influences on food choice are barely considered [28].

For physical activity, the EUropean-Physical Activity Determinants (EU-PAD) framework was developed [39]. The WP2.2 research team identified a list of 183 factors based on both expert judgement and empirical evidence. The concept mapping resulted in six distinct clusters, broadly merged in two themes: 1) the ‘Person’, which included the clusters ‘Intra-Personal Context and Well-being’ and ‘Family and Socioeconomic Status’ (42% of all factors), and 2) the ‘Society’, which included the remaining four clusters ‘Policy and Provision’, ‘Cultural Context and Media’, ‘Social Support and Modelling’, and ‘Supportive Environment’ (58% of all factors). Overall, 25 factors were rated as the most modifiable and impactful in terms of physical activity behaviour across the life course. They were largely situated in the ‘Intra-Personal Context and Well-being’ cluster [39].

For sedentary behaviour, the Systems of Sedentary behaviour (SOS) framework was developed [40]. The resulting framework maps the 190 potential factors in a system of six interacting clusters: ‘Physical Health and Well-being’, ‘Social and Cultural Context’, ‘Built and Natural Environment’, ‘Psychology and Behaviour’, ‘Politics and Economics’, and ‘Institutional and Home Settings’. In addition, priorities were set in terms of focusing research on the most potentially modifiable and impactful parts of the system. Investigating the influence of ‘Institutional and Home Settings’ was deemed to be the most promising area [40].

In the determinant framework developed specifically for ethnic minorities populations, seven distinct clusters emerged for dietary behaviour (containing 85 factors) and eight for physical activity behaviour (containing 183 factors). Four clusters revealed themselves to be similar across all behaviour: ‘Social and cultural environment’, ‘Social and material resources’, ‘Psychosocial’, and ‘Migration context’. The WP2.4 framework was aligned with those of the other three WPs. In general, the clusters of factors that emerged in the ethnic minority determinant framework were somewhat similar to the majority population frameworks for diet and physical activity behaviour, with the exception of ‘Migration context’. The importance of factors across all clusters was acknowledged, but their relative importance, or manifestation, differed for ethnic minority versus the majority population [42].

#### Secondary data analyses

A data pooling taskforce that spanned the WPs in TA1 and TA2 was established to develop a strategy for secondary data analysis. This group devised a five-step methodology covering 1) the identification of relevant datasets across Europe; 2) the development of a dataset compendium that included details on the design, study population, measures, and level of accessibility of data from each study; 3) the definition of key topics and approaches for secondary analyses; 4) the acquisition of access to datasets; and 5) the development of a data harmonisation platform, and pooling and harmonisation of the data [43]. Based on this, a variety of approaches to secondary data analysis were identified, including re-analysis of a single, existing dataset, ‘federated’ meta-analyses of two or more datasets based on a common data-analytical syntax applied to locally stored data, and the pooling, harmonisation and re-analyses of multiple datasets. To assist with these analytical approaches, a two-day statistical analysis workshop was organised in Amsterdam, the Netherlands, that specifically focused on the challenges associated with conducting secondary data analysis, handling pooling and harmonisation issues, and the provision of support in the area of advanced statistical techniques (e.g., federated data analyses, Bayesian analyses, mediation/moderation analyses, and handling missing data). In addition, WP2.2 and WP2.3 held a combined, three-day writing retreat in Ghent, Belgium to further define approaches, make progress on specific questions, as well as identify unresolved issues regarding the pooling and harmonisation process.

Critical aspects of the FAIR principles of data management and stewardship [44] were monitored throughout the phases of the secondary data analyses, from the identification of potentially relevant datasets to the actual reuse of data. The FAIR principles suggest that each data resource, associated metadata, and complimentary files should be easy to find (‘Findable’); provide relevant metadata from these datasets, on the types of variables, age groups under study, study design, measurement instruments used, time frame, etc. (‘Accessible’); be ‘Interoperable’ and thus use a consistent data format and taxonomy for knowledge representation; and, finally, they should be ‘Reusable’, i.e., made available for further analyses.

The topics and general outcomes of the secondary data analyses are briefly summarised below. We found that many different approaches to handling the multiple data sources were used, as were a wide variety of methods of statistical analysis. Regarding determinants of dietary behaviour, a total of 14 research questions have been or are currently being addressed using secondary and/or federated meta-analysis [45–50]. The published articles are listed in Table 2.

Compiling the physical activity and sedentary behaviour data resulted in a detailed list of 150 datasets. A total of 14 of these datasets were eventually obtained and reused to address 10 exemplar research questions on determinants of physical activity and sedentary behaviour (Table 2 contains the published papers). So far, these manuscripts have relied on a variety of methods of analysing the data, such as Bayesian Network analysis of the determinants of physical activity and sedentary behaviour provided by the Eurobarometer dataset, meta-regression to examine more complex interactions with selected moderator/mediator variables of physical activity behaviour within harmonised datasets, and Chi-squared automatic interaction detection (CHAID) to examine the hierarchy of socio-demographic correlates of remaining sedentary for an extended period of time [51]. The latter analyses included over 27,000 participants and showed that current occupation was primary discriminator. A deeper profiling revealed that highly educated adults with white-collar jobs, who had no difficulties paying bills, and used the internet frequently were most likely to sit too much.

In WP2.4, due to the limited availability of data on behaviour and determinants across ethnic minorities, specific case studies and secondary data analysis on single datasets were conducted, as opposed to federated meta-analysis or pooled analyses [52]. In general, these efforts revealed a clear lack of indicators of ethnic minority status in the studies that were included in the compendium. Furthermore, quite often the numbers of ethnic minorities included in some of the pan-European studies were too small to enable detailed analysis of specific groups, thus they are often ‘lumped’ together to increase statistical power.

The DEDIPAC data warehouse proved to be useful for pooling datasets, but in general, the available data -or rather the lack thereof- often restricted harmonisation to just a few core (crude) outcome variables and some individual-level, socio-demographic correlates of these behaviours. The stepwise approach to secondary data analysis used was described in a ‘methods’ paper [43], as well as in a position paper that draws on the possibilities and impossibilities of secondary data analyses of pooled and harmonised data on determinants of sedentary behaviour. The main gaps identified were lack of datasets that specifically emphasise determinants of behaviour - especially at the more macro level and with a systems approach; too few longitudinal studies examining determinants; and inadequate coverage of European nations and age groups. Most studies investigated the behaviour-disease relationship rather than the determinants of the behaviour.

### Objectives, methods, and results of TA3

TA3’s overall objective was to contribute to better evaluation and benchmarking of public health interventions and policies related to dietary, physical activity, and sedentary behaviour across the life-course. The specific goals of TA3 were:

1. To improve the quality of public policies and intervention to promote healthy diet, physical activity, and to reduce sedentary behaviour by creating a database with examples of good practice;
2. To improve implementation (from research to practice/policy) and transferability (from practice/policy to practice/policy) of public policies and intervention;
3. To develop and pilot test an online toolbox for developing, monitoring, and evaluating policies and multi-component interventions across Europe.

The work within TA3 was organised into three WPs; the first aimed to realise goals 1 and 2, and the two other WPs focused on the development and pilot testing of the toolbox (goal 3) - one focused on policies, and the other on multi-component interventions. ‘Multilevel or multi-component interventions’ were defined as theory-based interventions that use knowledge of behavioural determinants at different levels (i.e. individual, socio-cultural, and environmental) to improve dietary, physical activity, and sedentary behaviour in individuals. The focus was specifically on interventions that combined individual-level components with contextual or environmental-change components, as earlier evidence suggested that such combinations are most likely to be effective [53–55]. These interventions have not yet been widely implemented, but have the potential to be translated into health-promoting policies if adopted by governmental agencies in the future. They may thus be regarded as feasibility/pilot interventions to inform future policy making.

#### Improving quality of public policies and interventions

To contribute to meeting the first objective, an umbrella review (a review of reviews) was conducted first. This umbrella review focused on identifying good-practice characteristics for interventions and policies that aim to promote healthy diets and physical activity, and included systematic reviews, as well as position papers [56]. In this review, 53 good-practice characteristics were identified. Eighteen of these characteristics were related to intervention and policy content and focus, and were related to the use of theory, target populations and target behaviour, content development and management, multidimensionality, and practitioners and settings. Another 18 characteristics were related to monitoring and evaluation and were related to such issues as costs/funding, outcomes and evaluation of effects, evaluation of reach, and participation. The other characteristics (*n* = 17) were related to implementation and concerned issues such as participation processes, implementation partnerships, training of practitioners, the use of existing resources, maintenance, adaptation processes, and transferability.

Next, a so-called ‘quick scan’ was conducted of relevant interventions and policies to identify potential good practices in ten countries involved in DEDIPAC. The ‘founders’ (*N* = 79) of the ‘good practices’ were subsequently approached and asked to complete an online questionnaire to retrieve information related to good-practice characteristics and to provide additional information regarding the main characteristics of the policy or intervention, monitoring and evaluation, and implementation. Finally, based on the ‘quick scan’ inventory of good practices, and the responses to the online questionnaire, an online database of examples of good practice in terms of public policies and multicomponent interventions (*N* = 52) was developed. The purpose of creating this database was to increase the use and knowledge of good practices in designing and implementing public policies and multicomponent interventions. A total of 44 examples of good practices of policies and interventions from eight European countries represented in DEDIPAC were included in the database. The database contains information on intervention characteristics (including aim, target population, and behaviour, among others), monitoring and evaluation efforts and accomplishments and implementation, sustainability, and transferability conditions. Most of the interventions in the database focus on children in school settings and address both diet and physical activity. This database is publicly available online (https://www.dedipac.eu/toolbox/) and is supported by a factsheet with background information to support the use and dissemination of the database among policy makers and health-promotion professionals.

#### Improving implementation and transferability of policies and interventions

To meet the second objective of TA3, an umbrella review was conducted to identify critical implementation and transferability conditions [57]. This review focused on documents aimed at generating empirical evidence and evidence-based recommendations regarding implementation conditions for policies and interventions targeting healthy diet, physical activity, or sedentary behaviour. For the purpose of this umbrella review, so-called ‘stakeholder documents’ were also considered eligible for inclusion and, as such, the data banks of publications from eight major stakeholders, like the World Health Organization, were searched for relevant documents. Eighty-three conditions that were relevant to successful implementation were identified and these were further grouped according to the RE-AIM (Reach, Effectiveness, Adoption, Implementation, Maintenance) framework [58]. Eight implementation conditions referred to reach in the target population; five addressed efficacy of implementation processes; 24 dealt with adoption issues by staff or institutions; 43 referred to consistency, costs, and adaptations made in the implementation process; and three addressed maintenance. The vast majority of the implementation conditions identified (73 of 83) were relevant in terms of both multi-component interventions and policies. Seven implementation conditions were policy-specific and related to the increasing complexities associated with coexisting policies/legal instruments and their consequences for implementation, as well as politicians’ collaboration in implementation.

Additionally, six example interventions and six policies pertaining to diet, physical activity and/or sedentary behaviour were identified in five DEDIPAC countries (Belgium, Germany, Ireland, Norway, and Poland). Face-to-face, semi-structured interviews were held with 40 stakeholders in an effort to ascertain the things that health promotion professionals and policy makers believe are important in terms of factors impacting adoption, implementation, maintenance, and transferability. Analysis of these case studies showed that active involvement of relevant stakeholders from the political, health and education sectors, as well as that of intervention/policy implementers, and good communication between coordinating organisations and the government, private organisations, and settings were important factors contributing to the successful adoption and implementation of both interventions and policies [59]. Additional factors included sufficient training of staff to ensure implementation according to existing intervention/policy protocols, and tailoring of materials to match needs and (language) skills and socio-cultural context of various target groups. The respondents also indicated that maintenance of implemented interventions/policies depended on whether or not they were embedded in existing or newly created organisational structures in different settings and whether or not continued funding was secured (which often depends on political support).

#### Development and testing of the online toolbox

In developing a preliminary toolbox for the development, monitoring, and evaluation of public policies and multicomponent interventions across Europe, so-called ‘rapid reviews’ were conducted on monitoring and evaluation of public policies and multicomponent interventions aiming to promote healthy dietary, physical activity and/or sedentary behaviour. Additionally, a template for systematically describing relevant policies and multi-component interventions including content, implementation conditions and main characteristics (e.g., aim, target population), and monitoring and evaluation was developed. This enabled the systematic inventory of standardised measures used to evaluate effects of policies or multi-component interventions (i.e. in terms of changes in determinants, behaviours, and health outcomes). It also enabled the systematic summary of procedures and measures related to economic evaluations, including the application of counterfactual methods to determine *ex post* effectiveness on the basis of quasi-experimental data [60], and process evaluation measures.

All the information and content derived from the reviews, interviews, and inventories were then combined into a preliminary toolbox designed to aid in the development, evaluation, and implementation of public policies and multicomponent interventions. The toolbox was implemented as an online, wiki-based platform and was pilot tested in two rounds by DEDIPAC partners and external stakeholders, including policy makers and practitioners, respectively. Face-to-face interviews, telephone interviews, or written comments using a standardised feedback form were used to obtain feedback on issues such as visual appearance, technical features, functionality, content of the toolbox, as well as questions about omissions, remarks, and suggestions for improvement.

The feedback provided was generally related to three issues. First, the online, wiki-based platform was regarded as being user *unfriendly*. Second, in many cases, the content was regarded as being insufficiently extensive and detailed. The third issue identified was the (lack of) ease of navigation. Based on this feedback, the platform was changed to a web-based format, the content was expanded and made more detailed and more examples were provided, and navigation changes were made. The second draft of the toolbox was then presented, tested, and discussed by DEDIPAC partners during the consensus meeting. At this meeting, additional comments and suggestions regarding further, final changes, improvements, and recommendations pertaining to the current version of the toolbox and its future use were made.

At this stage, the draft toolbox was applied to policy and multi-component activities that were already running or that were soon to be implemented or disseminated in different countries, to test and further enrich the toolbox. Stakeholders involved in these policies and interventions were asked to apply the toolbox and complete structured feedback sheets. The toolbox was tested in the context of five policies and six multicomponent interventions and feedback was provided by the relevant users. Finally, a meeting to reach a consensus on the content, appearance, accessibility, and user-friendliness of the toolbox was organised among the TA3 partners.

Overall, the stakeholder evaluation of the usefulness, feasibility, and applicability of the toolbox was positive; feedback provided indicated that the database part of the toolbox provided good examples of policy and multi-component interventions, and that the toolbox was helpful in terms of planning future policy and multi-component interventions and their evaluation.

Some critical feedback and suggestions for further improvements were provided as well. These concerned a need for additional content and a linkage to other, existing resources, including (other) websites with relevant information; further adaptation of structure, design and navigation throughout the toolbox; and inclusion of more examples, possibly also in other European languages besides English. Based on this feedback, final adaptations were made to the toolbox and it was made publicly available online ( https://www.dedipac.eu/toolbox/).

This version of the toolbox is divided into four sections. The first section, DEVELOPMENT, guides users through the process of developing a policy or multi-component intervention. Next, EVALUATION, proposes and explains guidelines and specific instruments geared toward evaluating policies and multi-component interventions. IMPLEMENTATION provides information on the process of implementation and/or process evaluation. Finally, the NATURAL EXPERIMENTS section offers practical examples of policies and multi-component interventions.

## Discussion

Over a three-year period, researchers from thirteen countries across Europe joined forces to establish DEDIPAC, the first joint action taken by the Joint Programming Initiative in an effort to foster pan-European research to contribute to more healthful diets and increased physical activity across Europe. This joint action built and pursued a common research agenda aimed at realising collaboration between various scientific disciplines to expand knowledge, develop new insights, and reduce research overlap.

The overall aim of DEDIPAC was to improve the infrastructure and methodology for research on, and gain more insight into the determinants of dietary, physical activity, and sedentary behaviour over the life course. The many outputs that were – and are being – generated, provide an overview of the state of the art, as well as suggestions for further research on multilevel determinants of dietary, physical activity and sedentary behaviour over the life course. The outputs also focus on the interrelation of these determinants, existing and available measurement methods that can be used to assess this behaviour and its determinants, and to inform and evaluate policies and multilevel interventions.

Concrete products developed in the context of DEDIPAC include determinant frameworks for dietary, physical activity, and sedentary behaviour, and comprehensive, open-access toolboxes that provide measurement methods applicable to this behaviour and its determinants, as well as methods and tools to develop, implement, and evaluate interventions and policies.

The main results of DEDIPAC show that the evidence base on determinants of diet, physical activity and sedentary behaviour - and thus on the causes of the causes of non-communicable disease - is fragmented. The series of systematic literature reviews assessed the available evidence on the current nature of dietary, physical activity and sedentary behaviour in Europe, as well as on factors that influence these behaviours over the life course. These revealed a great deal of variation in terms of assessment methods used and reported behavioural outcome variables, such that information on physical activity, sedentary and dietary behaviour across all of Europe is scarce, and such that studies in this area are difficult to compare and findings are difficult to assimilate. The disabling lack of knowledge exposed by the systematic reviews may well stem from the dearth of available data on determinants – at least in Europe. The various secondary data analyses that were undertaken in the context of DEDIPAC highlighted a lack of data that prevents closure of the knowledge gap. The federated meta-analyses, pooling, and harmonisation actions revealed that available studies have focused almost entirely on socio-demographic factors in isolation, and have not yet investigated more distal, contextual factors in the built, social and economic environments. Consequently, the available evidence regarding determinants and correlates of sedentary behaviour, for example, generally says more about *who* is sedentary than *why* people are sedentary. This evidence may help to decide *whom* to target with interventions and policies, but not *what* to target or *how* to do it. Moreover, the ‘renewed’ and widespread appreciation for systems thinking was reflected in the four frameworks that were developed within DEDIPAC. These frameworks focus on understanding how *clusters* of factors interact synergistically or antagonistically to promote or prevent certain behaviour. As such, this challenges the current practice of reducing determinants of health behaviour to discrete factors organised according to conceptual levels and conducting analysis using linear models, and raises the question of whether or not this advances our understanding of how to intervene effectively.

Regarding the benchmarking and evaluation of policy interventions to address dietary, physical activity, and/or sedentary behaviour, DEDIPAC revealed some best practice information and implementation conditions, as well as a few more detailed examples of policy evaluation. However, the main result was that policy evaluation and benchmarking in our field is in its infancy and needs to progress towards evidence-based policy making. In parallel with DEDIPAC, the INFORMAS network and methodologies were further developed as a global network of public-interest organisations and researchers aiming to monitor, benchmark, and support public and private sector actions to create healthy food environments and reduce obesity and non-communicable disease [61]. Part of INFORMAS is a so-called ‘Government Healthy Food Environment Policy Index’ (Food-EPI) that is comprised of a ‘policy’ component with seven domains on specific aspects of food environments, and an ‘infrastructure support’ component with seven domains geared toward strengthening systems to prevent obesity. A second interesting development in this field of policy evaluation and benchmarking is the NOURISHING framework [62], developed by the World Cancer Research Fund to highlight the areas in which governments need to take action to promote adherence to a healthy diet and reduce overweight and obesity. NOURISHING and INFORMAS are linked and work together, focusing on nutrition environments and policies. DEDIPAC aligned with INFORMAS to push for the application of the Food-EPI in different countries across Europe in order to gain experience with and further develop this instrument, and to actually carry out benchmarking of public policies across Europe. DEDIPAC further pushed for and contributed to initial steps to work towards a similar instrument for benchmarking and monitoring physical activity and sedentary behaviour environments and policies.

DEDIPAC succeeded in forging new collaborations across Europe to align research focus, infrastructure, and funding for this important topic to contribute to furthering behavioural nutrition and physical activity research, as well as translating this research into practice and policy. DEDIPAC can be regarded as a European joint-programming experiment designed to make better use of the limited resources for research by doing more things better together than each country could do separately. We believe that this experiment worked well in the context of DEDIPAC; the series of interrelated reviews, the stepwise generation of comprehensive models, the inventories of best practices, making use of, pooling and harmonising international data, as well as the development of toolboxes to disseminate state of the art research to wider audiences would not have been possible without a joint agenda and governance. Additionally, DEDIPAC worked as a true Knowledge Hub; it has created new collaborations, led to exchanges of people and knowledge, established networks, and resulted in (international) research grant proposals.

### Gaps to close

The systematic analyses of existing knowledge in the context of the various systematic literature reviews identified several gaps. First, the definitions used for the outcomes (i.e. dietary behaviour, physical activity) were not consistent, with many different types and terms being used. Second, a wide range of study designs, measurement methods, population groups, investigated determinants, and outcomes emerged from the analysed studies, making it difficult to evaluate and compare the evidence, and to draw definitive conclusions.

Regarding measurement, there is an urgent call for more objective methods. These do start to play a bigger role in behaviour assessment, particularly the measurement of physical activity and sedentary behaviour, but are still subject to limitations of their own. For instance, objective methods still suffer from an inability to assess specific domains of physical activity or sedentary behaviour, and their use can be constrained by price, logistical requirements for data, storage and processing. In addition, there are many devices available to make such measurements nowadays, but these devices also vary in terms of their validity. Therefore, at least for the foreseeable future, more traditional, self-reported data will still need to be collected adjacent to the data collected from mobile devices, for example, to appropriately capture important information on contextual factors affecting the behaviour. However, for surveillance purposes, if we are simply interested in prevalence estimates of adherence to physical activity recommendations, then such context may be less important.

Standardisation of assessment methods for determinants still needs special attention. Even simple determinants, such as education and occupation, are assessed and classified using a plethora of different methods. This impedes comparison between studies, as well as harmonisation and data pooling. Recruitment of population-representative samples, especially for population surveillance, is another challenge; non-response has become a greater and greater issue, as response proportions have decreased steadily over the last few decades.

Third, the most commonly used study design was cross-sectional. This means that the strength of the evidence produced by these studies in inherently limited due to the impossibility of assessing the direction of observed associations, and therefore of identifying the *true* drivers of health behaviour and their quantitative impact. The lack of high-quality longitudinal data on behaviour, as well as potential determinants, is arguably the most glaring research gap. Longevity of surveillance systems and cohort studies needs special attention, especially with regard to funding systems that only provide funding for up to five years. Keeping measurement methods standardised over time is paramount, but it is far from standard practice. There is an obvious tension between keeping methodology constant over time and advances in measurement methods, which might be solved by including new measures adjacent to existing ones in order to maintain longitudinal comparability, while simultaneously improving the surveillance system. Further, surveillance systems that include worn technology to assess trends over time are, with few exceptions, virtually non-existent. Although data pooling has been proposed as a promising way forward, the reality is much more sobering. Harmonisation between measures for both behaviour and determinants is incredibly challenging and often impossible. Hence, the establishment of a pan-European cohort and/or surveillance system focused on dietary, physical activity and sedentary behaviour, and their determinants seems the best way forward to take this research field to the next level.

Fourth, information on relationships between determinants, especially determinants at different socio-ecological levels within systems, are severely lacking. Fifth, there is a lack of studies on this behaviour and its determinants in ethnic minorities. Finally, there is a need to establish links between pan-European research and European Research Infrastructures (RI). One obstacle to establishing synergies between health research and surveillance, and RIs is that in many cases no provisions were made at the time of the creation of the resource to account for sharing/merging/linking with another research community. Therefore, in many instances, the benefits associated with these efforts can only be reaped in the future. As such, future effort will be necessary to make these resources sufficiently useable for surveillance, but also for the research community.

The frameworks developed highlighted areas of priority and modifiability within the dietary, physical activity and sedentary behaviour systems, specific to age groups, and also for ethnic minority populations. For instance, the ‘Supportive Environment’ cluster was considered to be the highest priority for research out of all the determinant clusters for physical activity. These findings, as well as those from the other DEDIPAC frameworks, support the suggestion, and call for, a shift in focus from individual responsibility, personal commitment, and lifestyle choices, to the influence of social and physical environment on overcoming barriers to healthy behaviour.

The efforts made in the context of the secondary data analyses on determinants of physical activity and sedentary behaviour faced considerable barriers across all FAIR domains: data resources, associated metadata, and complimentary files were often not easy to find (‘Findable’). Retrieving relevant metadata from these datasets, on the types of variables, age groups under study, study design, measurement instruments used, time frame, etc., for instance, was a painstaking process (‘Accessible’). A consistent data format and taxonomy for knowledge representation was generally lacking (‘Interoperable’), and, finally, only a limited number of datasets were eventually found to be ‘Reusable’, i.e., made available.

Extending beyond FAIR, the harmonisation of independent variables and outcome measures under study was often problematic. The DEDIPAC Compendium of Datasets indicates substantial variation in assessment methods and operationalisation of variables across current European studies. This variation not only hampered the practical harmonisation process, but also presented comparability issues, as estimations of physical activity and sedentary behaviour levels are known to differ based on the assessment method used. The secondary data analyses’ focus on ethnic-minority groups revealed that within current ‘mainstream’ research on the determinants of health behaviour in Europe, ethnic minorities are often either not included, or their numbers are too small to enable meaningful analysis.

Despite DEDIPAC’s significant theoretical and knowledge progress on behaviour and its determinants, the paucity of high quality, EU-wide data collected using standardised methodology on both the behaviour, as well as a wide range of potential determinants, in accordance a systems perspective, is a major barrier that slows this momentum. This data shortage undermines the progress towards informing policy and interventions to tackle key non-communicable chronic disease related to unhealthy behaviour.

### Future directions

Regarding policy benchmarking and monitoring, we recommend the further development of the toolbox for policy evaluation and benchmarking by developing and testing an instrument for physical activity and sedentary behaviour similar to the INFORMAS food-EPI; combining and applying this alongside the food-EPI in different countries across Europe to gain hands-on experience with this type of policy monitoring and benchmarking across Europe. This will contribute to the further validation of the methodology, the dissemination of best practices across Europe, and thus to working towards better-informed, evidence-based policy making across Europe.

DEDIPAC was one of the first actions taken in the context of the Joint Programming Initiative in the field of behavioural nutrition and physical activity in Europe. This joint-programming experiment provided better overviews of the field, initiated new collaborations, created new insights, as well as identified directions for further research. DEDIPAC works toward and seeks support for three ways of building upon DEDIPAC to further the field:

1. Sustain and further strengthen the Knowledge Hub in an effort to create a sustainable European network centre for research and expertise on behavioural nutrition and physical activity, which could work under the umbrella of the International Society for Behavioural Nutrition and Physical Activity;
2. Provide longitudinal data on the individual and contextual drivers of the behaviour causing or aggravating non-communicable disease by building a prospective cohort of families across all regions of Europe, making use of the rich diversity in the systems of policies, contextual and individual determinants, as well as the behaviour across Europe from an early age onward. Analysis of the data acquired from this cohort should be subjected to harmonised methodology and measures, and focus on policy, contextual, as well as individual determinants of dietary, physical activity and sedentary behaviour from a life-course perspective. In addition, the cohort should be representative of the whole European population, including those who have migrated from other parts of the world;
3. Build a strong framework for monitoring, evaluation, and benchmarking of dietary, physical activity, and sedentary behaviour policies and environments across Europe.

## Conclusions

Europe has the right ingredients for the creation of infrastructure to study the causes of the causes of the chronic disease burden. DEDIPAC has strengthened existing infrastructure by aligning countries, research centres, and scientists from various disciplines across Europe on this crucial topic. It provided further insights into the measurement, the wide range of determinants of dietary, physical activity and sedentary behaviour across the life course and their interplay. However, action is now needed to build on this momentum. At present, we need better cross-European harmonisation of measurement and monitoring, FAIR data management and data sharing, common methodology, as well as (longitudinal) data required to gain more insight into behavioural determinants, as well as policy evaluation and benchmarking. We lack complete data on all of Europe, with data on southern and eastern European regions, in particular, being scarce.

## Abbreviations

CHAID: Chi squared automatic interaction detection
COSI: Childhood obesity surveillance initiative
DEDIPAC: Determinants of diet and physical activity
DONE: Determinants of nutrition and eating framework
ESFRI-BMS: European strategy forum on research Infrastructures- Biological and medical science
EU: European Union
EU-PAD: European physical activity determinants framework
FAIR: Findable, Accessible, Inter-operable, Reusable
FFQ: Food frequency questionnaire
Food- EPI: Government healthy food environment policy index
HBSC: Health behaviour in school-aged children
HDHL: A healthy diet for a healthy Life
INFORMAS: International Network for Food and Obesity/ non-communicable Diseases Research, Monitoring and Action Support.
JPI: Joint programming initiative
PRISMA: Preferred Reporting Items for Systematic Reviews and Meta-Analyses
PROSPERO: International prospective register of systematic reviews
RE-AIM: Reach, Effectiveness, Adoption, Implementation, Maintenance
RI: Research infrastructure
SIM: Systematic interdisciplinary mapping
SOS: Systems of sedentary behaviour framework
TA: Thematic area
WHO: World Health Organization
WP: Work package
